# Big Advocacy, Little Recognition: The Hidden Work of Black Patients in Precision Medicine

**DOI:** 10.21203/rs.3.rs-2340760/v1

**Published:** 2023-03-20

**Authors:** Lynette Hammond Gerido, Kenneth Resnicow, Elena Stoffel, Tiah Tomlin, Robert Cook-Deegan, Melissa Cline, Amy Coffin, Jill Holdren, Mary Anderlik Majumder, Zhe He

**Affiliations:** University of Michigan School of Public Health; University of Michigan School of Public Health; University of Michigan Rogel Cancer Center; My Breast Years Ahead; Arizona State University; University of California, Santa Cruz; Virginia Commonwealth University; The Light Collective; Baylor College of Medicine; Florida State University

**Keywords:** Breast cancer, Genetic testing, Advocacy, African Americans, Biomedical research, Data ethics

## Abstract

Public health genomics prioritizes effective and ethical translation of genomic science into population health precision medicine. With the rapid development of cost-effective, next-generation genome sequencing, calls are growing for greater inclusion of Black people in genomic research, policy, and practice. Genetic testing is often the first step in precision medicine. This study explores racial differences in patient concerns about genetic testing for hereditary breast cancer. Employing a community-based participatory mixed methods research design, we developed a semi-structured survey that was shared broadly. There were 81 survey respondents, of which, forty-nine (60%) self-identified as Black, twenty-six (32%) indicated they had a history of a breast cancer diagnosis, or had received BRCA genetic testing. Black participants who expressed concerns about genetic testing were fairly equally distributed between concerns that could be addressed with genetic counseling (24%) and concerns about the subsequent use of their genetic data (27%). The concerns expressed by the participants in our study underscore a need for transparent disclosures and assurances regarding the use and handling of genetic data. These findings should be viewed in context with patient-led efforts to overcome systemic inequities in cancer care, as Black cancer patients have joined forces with advocates and researchers to develop protective health data initiatives and to improve their representation in genomic datasets. Future research should prioritize the information needs and concerns of Black cancer patients. Interventions should be developed to support their hidden work as a means to reduce barriers and improve representation in precision medicine.

## Introduction

Public health genomics prioritizes the effective and ethical translation of genomic science into population health benefits and personalized health care. Genetic testing is often the first step in the process to personalize medicine. However, many cancer patients face barriers to accessing genetic testing and counseling services (Khan et al., 2022), and Black cancer patients have more pronounced disparities in survival. Disparities will widen (Huey et al., 2019) and personalized medicine cannot reach its full promise if barriers prevent many from benefiting.

Genetic testing has been available for many hereditary cancers since the 1990s. For Black patients in the United States, hereditary breast, ovarian, and colorectal cancers highlight stark disparities. Hereditary breast cancer and ovarian cancer (HBOC) incidence rates tend to be higher among younger (< 45 years old) Black women than among white women and mortality from breast cancer is 42% higher in Black patients than in white patients. Similarly, hereditary colorectal cancer tends to present earlier in Black patients than in white patients and Lynch syndrome is the most common colorectal cancer syndrome among high-risk Black patients (Garland et al., 2021). Black hereditary cancer patients are less likely to be referred to genetic testing and counseling services (Chapman-Davis et al., 2021; Dharwadkar et al., 2022; Graves et al., 2011; Sheppard et al., 2014).

Racial disparities in precision medicine often are a result of limited access to and awareness of clinical genetic evaluation services (Kaphingst et al., 2016; Krakow et al., 2017). Many interventions have been developed to address the disparity in breast cancer mortality between Black and white patients (Copeland et al., 2018), and Black breast cancer survivor-advocates play a critical role in addressing disparities (Jackson et al., 2021; Lythcott et al., 2003). Advocacy, which includes providing different types of support, improves self-advocacy and health-protective behaviors for the networks in which Black patients are embedded (Molina et al., 2016).

Initiatives to engage underrepresented populations in cancer clinical trials and genomic research are growing (Ramirez & Thompson, 2017; Saulsberry & Olopade, 2021). Although physician referrals and patient awareness are important, sociocultural experiences should be considered as well. Medical mistrust is more pronounced among Black patients (Powell et al., 2019; Sutton et al., 2019). Mistrust is often a byproduct of racial and ethnic discrimination, which may impact medical decision making and health behaviors. Schumann and colleagues (2021) found that mistrust may articulate critique of the profit-making with patient data (Schumann et al., 2021). Few studies reconceptualize mistrust of genetic testing through patient narratives. Many studies reference the historical traumas from the infamous Tuskegee Syphilis study to provide critical context, but this type of framing reference is overly simplistic and fails to account for many modern-day information technology concerns. Therefore, the goal of this study is to explore the concerns and experiences of Black breast cancer patients related to genetic testing for HBOC and to describe how those concerns relate to the hidden work of patient advocates.

## Method

### Study Design

This study employed a community-based participatory mixed methods research design. It was conducted in partnership with three breast cancer advocacy groups My Breast Years Ahead, a support group created by Black women to support others with breast cancer and affiliated with My Style Matters; Brave Bosom, a citizen data science organization affiliated with The Light Collective; and Facing Our Risk of Cancer Empowered (FORCE), a nonprofit organization for people with hereditary breast, ovarian, and other cancers. The first author conducted extensive pre-study fieldwork by attending meetings held by the advocacy groups and volunteering locally at events hosted by the Florida Breast Cancer Foundation (FBCF). Two other authors hold leadership roles within breast cancer advocacy groups [TT and JH] and were a critical bridge to understanding concerns of HBOC patients throughout the study. As a result of the fieldwork, we determined our research priority would be to better understand racial differences in concerns about genetic testing for hereditary breast and ovarian cancer (HBOC). The research was conducted in Tallahassee, Florida, as part of a PhD dissertation in Information Science. The study procedures were approved by the Florida State University Institutional Review Board.

### Sample

Adult participants, aged 18 or older, were recruited through snowball sampling. An invitation to participate in the study, including an anonymous link to the Qualtrics secure online survey software, was distributed on social media outlets and online via cancer advocacy groups’ web posts or newsletters, was posted on social media sites with messaging to encourage readers to share the invitation with others. Cancer advocacy groups organized around inherited cancer risk constituted a natural constituency. Surveys were considered complete if more than 80% of the eligible questions were answered. For their time and effort, all participants who completed the survey received a $10 Amazon gift card.

Out of 102 surveys submitted, 81 were complete. The respondents ranged in age from 24 to 76 years. All resided in North America. The sample was stratified by race (Black, Asian, White, and Other). Black women with breast cancer were deliberately targeted for recruitment. Forty-nine respondents (60%) self-identified as Black. Twenty-six (32%) indicated they had a history of a breast cancer diagnosis.

### Data Collection

The data were collected using a secure online survey, which included structured items and unstructured free text questions, adapted from validated measures in the Health Information National Trends Survey (HINTS) (Health Information National Trends Survey ∣ HINTS, n.d.). The National Cancer Institute’s HINTS is a nationally representative cross-sectional survey administered biennially to adults aged 18 years and older in the United States to monitor health information awareness and communication (Hesse et al., 2017). We refined the instrument with input from the advocates to include an open-ended qualitative question about genetic testing concerns. Data collection took place between March and November 2020. Both quantitative (structured) and qualitative (unstructured, free text) data were collected.

### Measures

We collected the following socio-demographic information: self-reported race, ethnicity, age, education, income, marital status, insurance, and geographic region. Cities where the participants currently reside were grouped into geographic regions. Questions to capture a brief health history were included self-reported history of a breast cancer diagnosis and family cancer history were included. We collected information about the participants’ awareness of genetic tests in general and their past experience with genetic testing. In addition, participants were asked about their willingness to take a genetic test and we captured information about genetic counseling. Genetic testing concerns were captured as an unstructured field, in which participants were asked to type a response to the question “Do you have any concerns about genetic testing? Please describe.”

### Data Analysis

Prior to analysis, all responses were deidentified and assigned a unique participant number. We conducted a complete case analysis to screen for errors, determine frequencies, and identify normality of distribution patterns in the data to assess its overall quality. We used R to conduct exploratory data analysis and preprocess the data.

Descriptive statistics were calculated for all variables of interest. Continuous measures were summarized using means, standard deviations, and ranges. Categorical measures were summarized using counts and percentages.

### Thematic Analysis

We created a preliminary unstructured coding guide based on constructs of Dervin’s Sensemaking Theory (Dervin, 1998). Sensemaking theory provides a framework for identifying situations, recognizing barriers and social contexts, describing gaps or discontinuities, and creating bridges to desired outcomes. We introduced six undergraduate research assistants (URAs) to coding unstructured data using the coding guide. During a weekly lab meeting the URAs and the first author reviewed 10 random responses to the unstructured question capturing genetic testing concerns. Line by line data coding was then conducted collaboratively to assist in clustering of responses and refining the codebook. As the URAs reviewed each response, they associated it with items in the preliminary coding guide or refined the guide with new codes as required. Codes were compared and discussed until we arrived at a consensus. The final code structure contains 8 codes, which we subsequently categorized under two overarching themes; concerns addressed with genetic counseling and concerns about the subsequent use of genetic information.

## Results

### Participant Sociodemographic and Background Characteristics

All participants identified their gender as female or gender nonconforming. The study population was educationally diverse, with higher educational achievement than the general population (18.5% with high school diplomas, some college, or an Associate’s degree; 30.9% with a Bachelor’s degree; and 48.1% with a Master’s degree or higher). The study population reported 28.4% below $60,000, 34.6% between $60,000 and $99,000, and 27.2% with $100,000 or more annual household income. Most had insurance (93.8%), were married (48.1%), and had children (76.5%). Only 8.6% indicated Latinx or Hispanic ethnicity. Black participants represented 60.5% of the study population.

### Racial Differences in Concerns

The frequency of the two themes (concerns addressed with traditional genetic counseling and concerns about subsequent use of genetic information) between Black participants and non-Black participants showed interesting differences (Table 1). Nearly half (49%) of the Black participants expressed no concerns with genetic testing, in comparison to fewer non-Black participants who expressed no concern (28%). Black participants with concerns were fairly equally distributed between concerns that could be addressed with genetic counseling (24%) and concerns about the subsequent use of their genetic data (27%).

Non-Black participants shared more concerns that could be addressed with genetic counseling (47%) than concerns related to the subsequent use of their genetic test data (25%). Both Black and non-Black participants expressed similar concerns that are described in the following passages, which include exemplar quotations.

### Theme I: Concerns Addressed in Traditional Genetic Counseling

There were five unique concerns expressed by participants that traditionally have been addressed by Genetic Counselors: validity of results, medical decisions, education, insurance considerations and family communications.

### Validity

Participants reported concerns about the

“possibilities of inaccuracies”

(P03)

in the test results. This implies that patients prefer certainty in the results. Black breast cancer patients who have undergone genetic testing often receive uncertain results or VUS (variants of uncertain significance) results, which reflects an ever-evolving need for research and discovery related to the classification of variants. Populations with African ancestry are underrepresented in genetic databases that classify variants and genetic testing results can vary in clinical validity.

### Medical Decisions

Genetic testing results are important tools for making medical decisions. At various stages along the breast cancer care continuum, patients can feel unsure how to make decisions based upon the results of the genetic test.

“My concern is when they find an anomaly and there isn’t any information in how to deal with it.”

(P50)

Some may opt to be tested before breast cancer is suspected because they have a family history of HBOC or perhaps they engaged in recreational testing purely for the knowledge. Others may be recently diagnosed and need to use the information to make surgical decisions to prevent the progression of the disease. While others may have survived breast cancer and have incorporated genetic testing as a means of surveillance in case of recurrence. In each of these situations, the genetic testing would address different types of medical decisions.

### Education

Patients expressed a need to be educated about the process and what to expect after the testing has been completed.

“Honestly I probably don’t know enough about the testing to really know if I should be concerned.”

(P65)

Patients may need information in order to raise questions and self-advocate.

### Insurance

Insurance concerns were expressed by the participants. They worried about

“Insurance ramifications”

(P08)

The Genetic Information Nondiscrimination Act of 2008 helps to prevent health insurance plans discriminating against patients with genetic susceptibility for diseases and disorders. However, these types of concerns may be extremely important for marginalized groups due to increased likelihood of encountering structural barriers to care and limited insurance benefits. Some patients may be reluctant to agree to genetic testing due to perceived changes in medical costs or reduced insurance coverage.

### Family Communications

When patients receive the news that they have a hereditary cancer syndrome, it can impact the entire family. Patients who have a pathogenic variant often carry the primary responsibility to communicate directly with relatives, which can be especially difficult for parents.

“I do [have concerns] for my own children - not sure when is too early.”

(P43)

Standard practice is for providers to support patients with “a family letter” to share the news of their genetic risk and to encourage them to seek genetic testing and counseling services as well.

### Theme II: Concerns about Subsequent Use of the Genetic Information

Concerns about how the genetic information would be stored, kept private, or destroyed after testing were described. Participants wanted to know who would have access to the information and whether it would be used to socially discriminate against them or to commit genocide.

### Agency

In addition to using genetic testing results to make medical decisions, patients would like to have agency in what happens to their genetic information.

“After the testing what happens to my genetic samples? Will they be kept, disposed of, or returned to me? Am I able to make that decision?”

(P39)

### Privacy

Concerns about agency are related to protecting the privacy of such sensitive information. Truly protecting privacy can be a challenge with de-identification.

“Information security, privacy, testing or any part of the data analysis being done outside the U.S.”

(P56)

Genetic data is unique to the individual and even if it has been separated from other personally identifiable information, genetic data is forever linked to the individual. People often view genetic information about themselves as private. Specific variants within an individual’s genome may be widely shared with biological relatives or even across the entire human population.

### Third Party Access

Often laws limit how genetic data may be used or by whom the data may be accessed but patients are not aware of how their genetic data will be protected.

“I learned after the test about potential problem of obtaining life insurance because of genetic testing regardless of the results. I feel those who are tested should be made aware of that fact.”

(P29)

### Social Discrimination

Some marginalized communities have faced historic trauma related to discrimination and oppression, which bleeds into current day experiences with social discrimination.

“My concern would be if my results could be used towards other testing or sold to outside companies.”

(P47)

Black breast cancer patients may experience disproportionate exposure to forensic surveillance and overrepresentation in law enforcement genetic databases. Consideration of the additional dimensions of discrimination may be a priority among Black patients.

### Genocide

Genocide is the deliberate killing of people related by geographic region, ethnicity, religion, national origin, or other physical or genetic characteristic. This may sound like a farfetched concern but historically there have been a number of genocidal acts in the 20th century, including the Holocaust which is the largest and most well documented case of genocide in human history.

“Just worry future Nazi genocidal maniacs will use the information against us.”

(P79)

The historical ties between human genome research and antiquated eugenics practices may foster additional concerns that extremist organizations will use genetic information to carry out genocide.

## Discussion

This study demonstrates the value of partnership with underrepresented communities in public health genomics research and expands our awareness of breast cancer advocacy efforts. When one thinks about breast cancer advocacy, typically the efforts are seen through a lens of social support. Many advocates address barriers related to social determinants of health by providing transportation to appointments, raising funds for housing, and offering healthy meals. Yet there are additional collective advantages of having advocacy groups embedded within research teams. Advocacy extends beyond the clinical research settings into underrepresented communities. From the outset of this study, partners from underrepresented communities affected by cancer and specifically those that supported Black breast cancer patients were engaged. This aided in successfully representing Black women in our study population.

Our study is novel in that nuances of medical mistrust have been unpacked as patient concerns specific to genetic testing. We discovered two categories of concerns expressed about genetic testing, one theme reflecting a need for genetic counseling prior to testing and the other reflecting issues specific to genetic data use after a test has been taken. Although Black participants in this study raised concerns about genetic testing that are similar to those raised by non-Black participants, their data use concerns may highlight an imminent need. We must increase access to genetic counseling services in parallel with more transparent disclosures and assurances about the use and handling of genetic data. These findings should be viewed in context with existing patient-led efforts to overcome systemic inequities in cancer care. Advocacy groups have become an ongoing source of emotional support and a bridge to activism and representation (Braun, 2003). Black breast cancer patients have been partnering with advocates and researchers to educate communities, develop protective health data initiatives, and to improve the representativeness of genomic data for quite some time.

### Genetic Counseling and Education

Our study shows that among Black participants who expressed concerns about genetic testing, nearly half of those concerns could be addressed by having access to genetic counseling services. Typically, genetic counseling helps patients make informed, autonomous choices about whether or not to undergo a genetic test, how to interpret the results, and weigh options related to the results. Medical decisions can be challenging and genetic testing can further the complexity of considerations. For example, advocates have developed extensive educational materials to describe the differences between direct-to-consumer genetic testing (i.e., 23andme) and genetic testing provided by laboratories regulated by the Clinical Laboratory Improvement Amendments or CLIA (i.e., Color Health). These educational materials are designed to help patients understand the differences in testing technologies, the limitations of the results, and the potential data risks related to the subsequent use of their genetic data. This work is significant for Black breast cancer patients, who may require CLIA-certified laboratory tests because to identify more than the three Ashkenazi Jewish founder variants. Also, relevant to patient concerns about data use, the CLIA-certified laboratories are not able to resell genetic testing data. Advocates make these types of educational materials accessible to supplement information from health care providers. This is an example of information that would be discussed as a part of genetic counseling.

Unfortunately, there is a clear shortage of health professionals specifically trained to provide genetic counseling. Advocacy groups fill these voids and unmet needs of communities vulnerable to lacking access to genetic counseling. Without access to certified genetic counselors, many patients facing a hereditary cancer diagnosis seek information and decision support from trusted advocacy groups (Davis & Baca, 2015). Many advocacy groups like My Breast Years Ahead disseminate educational materials on behalf of healthcare providers and direct vulnerable patients to additional resources. Advocacy groups assist patients in improving their communicative competence with providers and their family members.

### Data Use and Governance

Our findings indicate that Black participants are interested in genetic testing but they have reservations about the misuse and abuse of genetic data. The technologies and data structures, which support clinical genetic services must be evaluated and updated often. Recent news headlines describing the racial inequities built into clinical algorithms used for monitoring and surveillance can erode trust in clinical technologies. “…we are in need of new analytic tools, forged by critical scholars of science and technology, that draw into view the mutual constitution of social and scientific practice”(Reardon, 2008). We must reimagine equity and justice in healthcare and recognize that systems architectures can engineer inequality (Benjamin, 2016). We can reconnect with the efforts of geneticists, like Mary-Clare King, whose activism and social justice agenda bridged scientific and sociopolitical spheres (Nelson, 2016).

Communities, patients, and advocates need to be embedded within interdisciplinary teams that identify variants and build systems that use their data. Co-creation of knowledge and systems is required to build and sustain trust with diverse communities. Data trusts may facilitate the use of data across organizational boundaries and protect the interests of multiple stakeholders (Gomer & Simperl, 2020) by empowering patients with control over their personal data (Raab, 2021). The Light Collective is an advocacy group that works with breast cancer and other online patient peer support communities to develop models of collective data governance where patients control how their data are used, by whom, and for what purpose. Future research should offer opportunities for advocates to engage in research intended to govern the subsequent use of genetic information as well as offer adequate financial support and resources for existing educational outreach activities. We must recognize the invisible labor of breast cancer advocacy groups that currently serve the underserved and underrepresented communities.

### Strengths and Limitations

Racial disparities in the uptake of genetic testing for breast cancer consists of many factors. The results of this study add to the literature that intersects precision medicine and sociotechnical systems to advance medical care and reduce health disparities. Although this study was limited in scope and a small convenience sample, the findings challenge us to engage in new ways with genetic data. Future research should be conducted with a larger, less homogenous sample to verify the types of concerns, document the frequency at which they are present and generalizable in the population, and identify discordance within and between groups.

## Conclusion

Concerted interdisciplinary actions by multiple stakeholders are required to translate results into clinical practice. Data security and privacy protections are a priority. Both traditional genetic counseling and protective data initiatives are required to educate and build trust with underrepresented communities. Fostering meaningful partnerships with breast cancer advocacy groups may be the key to achieving representative genomic datasets and more equitable access to precision medicine.

## Figures and Tables

**Table 1:** Comparison of Themes by Race

	No Concerns	Concerns Addressed with Genetic Counseling	Concerns about Subsequent	Row Total
			Genetic Data Use	
Black	24 (49%)	12 (24%)	13 (27%)	49 (60%)
non-Black	9 (28%)	15 (47%)	8 (25%)	32 (40%)
Column Total	33 (41%)	27 (33%)	21 (26%)	81 (100%)
